# Association of Motorcycle Use with Risk of Overweight in Taiwanese Urban Adults

**DOI:** 10.3390/ijerph14040410

**Published:** 2017-04-12

**Authors:** Chien-Yu Lin, Yung Liao, Jong-Hwan Park

**Affiliations:** 1 Institute of Health Behaviors and Community Sciences, National Taiwan University, 17, Xuzhou Road, Taipei 100, Taiwan; r05850001@ntu.edu.tw; 2 Department of Health Promotion and Health Education, National Taiwan Normal University, 162, Heping East Road Section 1, Taipei 106, Taiwan; 3 Institute of Convergence Bio-Health, Dong-A University, 32, Daeshingongwon-Ro, Seo-Gu, Busan 49201, Korea

**Keywords:** sedentary behavior, motorcycle use, overweight, urban adults, transportation

## Abstract

Sedentary transport is known to adversely affect health. Few studies have focused on motorcycle use. This study examines the association of motorcycle use with overweight in urban adults in Taiwan. Cross-sectional data from 1069 Taiwanese adults aged 20–64 years in three urban cities were collected in 2015. Data on self-reported body mass index, time spent in motorcycle use, lifestyle behavioral factors, and sociodemographic variables were obtained. Unadjusted and adjusted logistic regression models were applied. In Model 1, adults who spent more time using a motorcycle (third quartile, odds ratio (OR) = 1.17; fourth quartile, OR = 1.60) were more likely to be overweight compared with the first quartile. In Model 2, after adjusting for the covariates, only the fourth quartile of motorcycle use (OR = 1.50) was associated with a higher risk of overweight. Higher time spent in motorcycle use is related to higher risk of being overweight, even after adjustment for potential demographic and behavioral confounders. Intervention and behavioral change strategies targeting motorcycle use should be considered.

## 1. Introduction

Increased body mass index (BMI) is a major risk factor for non-communicable diseases such as cardiovascular disease, diabetes, musculoskeletal disorders, and certain types of cancer [1,2]. Despite these deleterious health impacts, an increasing worldwide prevalence of overweight and obesity has been reported [3], and it was estimated that approximately 13% of the world’s adult population were obese and 39% were overweight in 2014 [2]. Consistent with this trend, the prevalence of overweight (defined in Taiwan as a BMI ≥ 24 kg/m^2^, including obesity) in Taiwanese adults was estimated at 44.8% in 2015 [4] and has increased ~1.35-fold since 1996 [5]. Thus, identifying the behavioral risk factors associated with overweight is a public health priority in order to develop effective strategies for obesity prevention.

Choice of method of transport to and from destinations such as the workplace, home, and leisure facilities is usually a habitual behavior [6]. Research has indicated that active modes of transport (walking and cycling) and public transport (involving small bouts of walking and cycling) are both widely encouraged, mainly because they have several health benefits and are particularly associated with a lower BMI [7,8,9] compared with passive modes of travel. Sedentary transport is known to adversely affect human health and is thus not considered an active mode of transport [10,11]. Long periods of time spent sitting in private motorized vehicles have been associated with higher risks of weight gain [12,13,14,15]. However, while most previous studies have focused on time spent sitting in cars, few studies have examined specific forms of transport-related sedentary behavior, such as motorcycle use, especially since there is a particularly high prevalence of motorcycle use in Asian countries where motorcycles are affordable [16]. For example, Taiwan is a motorcycle-oriented country with a particularly high rate of motorcycle ownership (58.2 per 100 people in 2015, and consistent over the last three years) compared with other countries [17]. Nearly half of Taiwan’s adults use a private motorcycle as the main mode of commuting to work and traveling to other destinations [18]. Considering different residential densities and cultural factors in car-oriented countries as well as the deleterious health effects of sedentary transport on overweight [12,13,14], a better understanding of the association of the time spent in motorcycle use with the risk of overweight in Taiwanese adults would yield important information for public health initiatives. It was hypothesized that, after adjusting for covariates of sociodemographic and lifestyle behavioral factors (including physical activity, sleep, alcohol use, smoking status, and diet), dose-response associations might exist in the associations between motorcycle use and the risk of overweight. Therefore, this study examined associations of motorcycle use with the risk of overweight in urban Taiwanese adults.

## 2. Materials and Methods

### 2.1. Participants

Data in the present cross-sectional population-based study were obtained using a random-digit-dialing telephone survey conducted over a period of one month from September to October 2015. Detailed study methods and participant attributes have been previously reported [19]. As described in a previous study [19], a total of 5333 adults aged between 20 and 64 and living in one of these three cities—Taipei City, the capital of Taiwan (area 271.8 km^2^, population 2,704,810); New Taipei City (area 2052.6 km^2^, population 3,970,644); and Kaohsiung City, the largest and second city in Taiwan (area 2951.9 km^2^, population 2,778,918)—were eligible. Of the 5333 eligible adults, 1069 completed the survey (response rate: 20.04%). Respondents who completed the survey were offered no rewards but were asked for their verbal consent before the beginning of each telephone interview. The study protocols were reviewed and approved by the Ethics Committee of National Taiwan University (201504HM005).

### 2.2. Outcome Variable

The outcome variable of this study was the BMI calculated from self-reported height and weight (calculated as weight in kilograms divided by the square of height in meters) and dichotomized into normal weight (<24 kg/m^2^) or overweight (including obese, ≥24 kg/m^2^), according to the criteria set forth by the Ministry of Health and Welfare of Taiwan [20].

### 2.3. Motorcycle Use

The independent variable was self-reported motorcycle use, obtained by modifying the transportation items in the International Physical Activity Questionnaire-long version (IPAQ-LV) [21]. Respondents were asked, “During the last 7 days, on how many days did you travel on a motorcycle?” followed by, “How much time did you usually spend on one of those days traveling on a motorcycle?” The total time spent engaging in motorcycle use was calculated by multiplying frequency of motorcycle use (number of days in the previous 7 days) per week by duration of motorcycle use (minutes) per day. Because the distribution of motorcycle use was skewed, the respondents were categorized into quartile categories of motorcycle use—first quartile (0 min/week,) second quartile (1–69 min/week), third quartile (70–279 min/week), and fourth quartile (more than 280 min/week)—to examine the possible dose–response associations between motorcycle use and risk of overweight.

### 2.4. Car Ownership

The number of vehicles owned was recorded. Respondents were asked, “How many cars can be used to travel in your home?” The responses were categorized into “yes” (more than 0) and “no” (0).

### 2.5. Lifestyle Factors

Lifestyle variables included six related behaviors: transport-related physical activity (TPA), leisure-time physical activity (LTPA), sleep, alcohol use, current smoking status, and dietary behavior (fruit and vegetable consumption).

#### 2.5.1. Leisure-Time and Transport-Related Physical Activity

Both types of physical behaviors, TPA and LTPA, were obtained from the Taiwanese version of the IPAQ-LV. Test–retest reliability and criterion validity of the Taiwanese version of the IPAQ-LV have been confirmed [21]. For TPA, the second part of the IPAQ-LV was used to measure the frequency (number of days in the previous seven days) and duration (minutes per day) of engaging in “walking for transport” and “cycling for transport.” The total time spent engaging in transportation was calculated by multiplying frequency of transportation per week by duration of transportation per day.

For LTPA, the fourth part of IPAQ-LV was used to measure the frequency (number of days in the last seven days) and duration (minutes per day) that respondents had reported in moderate intensity of physical activity (MPA), vigorous intensity of physical activity (VPA), and walking during leisure time. The total time in leisure-time physical activity was calculated by multiplying the frequency of MPA, VPA, and walking per week with the duration per day. Total time spent in TPA and LTPA was dichotomized according to the categories in the public health guidelines [22] as sufficient (≥150 min/week) and insufficient (<150 min/week).

#### 2.5.2. Sleep

Sleep was measured by asking respondents, “How many hours in each 24 h day do you usually spend sleeping?” Fewer than seven or more than nine hours/day of sleep was coded “at risk” (i.e., short/long sleep durations), as reported by meta-analyses on sleep and health outcomes [23,24].

#### 2.5.3. Alcohol Use, Current Smoking Status, and Fruit/Vegetable Consumption

For alcohol use, respondents reported the total number of alcoholic drinks they consumed each week. The respondents were categorized into “Yes” and “No.” Regarding current smoking status, respondents were asked to report whether they were current smokers. In addition, respondents were asked how many servings of fruit and vegetables they usually consumed each day. Not meeting Taiwanese dietary guidelines for fruit and vegetable consumption (i.e., two servings of fruit and three servings of vegetables) was used as a marker for dietary risk [25].

#### 2.5.4. Sociodemographic Variables

The sociodemographic variables covered by the survey were gender, age, city of residence, education level, occupation type, marital status, and living status. These variables were further categorized as follows. Age was divided into five categories: (a) 20–29; (b) 30–39; (c) 40–49; (d) 50–59; (e) 60–64 years. Education level was categorized into (a) high school degree or lower (including elementary school, junior high school, high school, and vocational school degree) and (b) university degree or higher. Occupation type was divided into two categories: (a) part-time and (b) full-time. Marital status was divided into (a) married and (b) unmarried (including widowed, separated, and divorced). Accommodation status was divided into (a) living with others and (b) living alone.

### 2.6. Statistical Analysis

Data from 1069 Taiwanese urban adults who provided complete information for the study were analyzed. Pearson’s chi-square tests were used to distinguish the proportional differences in variables by different quartiles of groups. Forced-entry adjusted logistic regression was conducted to examine the associations of motorcycle use with risks of overweight. Two models were examined. Model 1 was an unadjusted model. In Model 2, the covariate of sociodemographic factors and six lifestyle behavioral factors were adjusted for. Adjusted odds ratios (ORs) and 95% confidence intervals (CIs) were calculated for each variable. Inferential statistical analysis was performed using IBM SPSS 22.0 software (IBM, Armonk, NY, USA), and the level of significance was set at *p* < 0.05.

## 3. Results

### 3.1. Participant Characteristics

Table 1 presents the participant characteristics (mean age: 45.1 years, standard deviation (SD) = 12.2). The average motorcycle use time was 26.20 min/day (SD = 38.23), and the median was 10 min/day. A percentage of 40.1% of respondents were overweight or obese. Those who spent more time using motorcycles were significantly more likely to be male, middle-aged, living in Kaohsiung city, and overweight.

### 3.2. Lifestyle Factors Associated with Motorcycle Use

Table 2 shows the associations between the lifestyle factors and motorcycle use. Among all participants, 44.8% had sufficient LTPA, 69.0% had sufficient TPA, 62.1% slept for appropriate durations, 10.5% used alcohol, 13.2% were current smokers, and 73.7% met the daily Taiwanese dietary guidelines. Those who spent more time engaging in motorcycle use were more likely to be current smokers and engaged in less TPA.

### 3.3. Motorcycle Use Associated with Overweight

Based on unadjusted analyses in Model 1, those who spent more time using a motorcycle were more likely to be overweight (see Table 3). The participants who spent more time using a motorcycle in the last seven days of the third quartile (OR = 1.17; 95% CI: 0.85–1.59) and the fourth quartile (OR = 1.60; 95% CI: 1.17–2.20) were significantly associated with higher risks of being overweight or obese compared with participants in the first quartile. While adjusted by the potential demographic and behavioral confounders in Model 2, only participants in the fourth quartile of motorcycle use (OR = 1.50; 95% CI: 1.06–2.13) were more likely to be overweight.

## 4. Discussion

The main finding of this study is that, after adjusting for six lifestyle behavioral risk factors (LTPA, TPA, sleep, alcohol use, smoking status, and dietary behavior), more time spent in using a motorcycle is associated with a higher risk of overweight. This finding is not only consistent with previous studies of car use [12,13,14] and private motorized vehicles [15], but it also extends the previous findings in that the use of another common mode of sedentary transport in Asian countries—the motorcycle—could also potentially increase the odds of being overweight. These results may have important implications for policy-makers or intervention designers to consider when developing effective strategies tailored to reduce overweight risk among motorcycle users, especially in motorcycle-oriented countries.

To our knowledge, few previous studies have examined the associations of motorcycle use with the risk of overweight. Taiwan is a motorcycle-oriented country with high motorcycle ownership and prevalence of overweight [5,17]. Although dose-response associations between motorcycle use and overweight risks have not been found in the present study, our results suggest that greater levels of motorcycle use could be a potential risk factor associated with overweight among Taiwanese urban adults. A possible explanation is that spending a greater time traveling by motorcycle may involve longer periods engaged in non-exercise activity (thermogenesis), which may be associated with increased risks of overweight [26,27,28] even though sedentary transport only occupies a small portion of total sedentary time [29,30]. Thus, future studies using prospective design are still needed to further examine the association between motorcycle use and weight gain.

The current study has several limitations. First of all, because of the cross-sectional design, we could not draw conclusions regarding the causal relationship between motorcycle use and risk of being overweight. Secondly, the main measurements including motorcycle use and BMI were self-reported and thus could be subject to bias [31]. Thirdly, there were several potential confounders, such as income, for which we could not adjust. In our past telephone surveys, a number of Taiwanese adults, especially those of low socioeconomic status, have refused to answer questions about income. Therefore, we excluded the question in this survey and could not consider it as a confounder. Fourthly, the diet behavior included in this study emphasized vegetable and fruit consumption; other diet habits, such as intake of high-glycaemic index (GI) foods, were not investigated. Finally, this study had a limited representative sample because it relied on a telephone-based survey; therefore, sections of the population without a household telephone (approximately 7.1% in 2015) were impossible to reach [32]. Our sample is also more highly educated (61.8% have a university degree or higher, compared to 43.6%) and more lower employed than the greater population (68.8% have a full-time job, compared to 95.9%) [33,34]. Thus, the findings of the present study may not be generalizable to the overall population.

## 5. Conclusions

Higher levels of time spent in a specific form of sedentary transport—motorcycle use—are associated with higher risks of being overweight, even after adjusting for potential demographic and behavioral confounders. Intervention and behavioral change strategies targeting motorcycle use should be considered.

## Figures and Tables

**Table 1:** Basic characteristics of respondents (*n* = 1069).

Basic Characteristics	Total	Frequency of Motorcycle Use				*p*
		Q1	Q2	Q3	Q4	
*n* (%)	1069	401 (37.5%)	108 (10.1%)	288 (26.9%)	272 (25.4%)	
***Gender***						<0.001 **
Men	526 (49.2%)	42.1%	46.3%	51.4%	58.5%	
Women	543 (50.8%)	57.9%	53.7%	48.6%	41.5%	
***Age (year)***						0.001 *
20–29	140 (13.1%)	13.2%	3.7%	11.8%	18.0%	
30–39	232 (21.7%)	19.0%	30.6%	20.5%	23.5%	
40–49	261 (24.4%)	20.9%	26.9%	26.4%	26.5%	
50–59	289 (27.0%)	31.4%	22.2%	26.7%	22.8%	
60–64	147 (13.8%)	15.5%	16.7%	14.6%	9.2%	
***Residential City***						<0.001 **
Taipei City	353 (33.0%)	47.9%	26.9%	23.3%	23.9%	
New Taipei City	364 (34.1%)	34.9%	39.8%	33.3%	31.3%	
Kaohsiung City	352 (32.9%)	17.2%	33.3%	43.4%	44.9%	
***Educational Level***						0.068
High school degree and lower	408 (38.2%)	33.4%	40.7%	43.1%	39.0%	
University and higher	661 (61.8%)	66.6%	59.3%	56.9%	61.0%	
***Occupational Type***						0.057
Not full-time	334 (31.2%)	34.9%	31.5%	31.9%	25.0%	
Full-time	735 (68.8%)	65.1%	68.5%	68.1%	75.0%	
***Marital Status***						0.069
Not married	351 (32.8%)	31.2%	26.9%	31.6%	39.0%	
Married	718 (67.2%)	68.8%	73.1%	68.4%	61.0%	
***Accommodation Status***						0.808
Living alone	52 (4.9%)	4.7%	6.5%	4.2%	5.1%	
Not living alone	1 017 (95.1%)	95.3%	93.5%	95.8%	94.9%	
***Body Mass Index***						0.030 *
Non-overweight	640 (59.9%)	64.3%	58.3%	60.8%	52.9%	
Overweight/obese	429 (40.1%)	35.7%	41.7%	39.2%	47.1%	

* *p* < 0.05; ** *p* < 0.001.

**Table 2:** Lifestyle factors associated with the frequency of motorcycle use among Taiwanese adults.

Lifestyle Factors	Total	Frequency of Motorcycle Use				*p*
		Q1	Q2	Q3	Q4	
*n* (%)	1069	401 (37.5%)	108 (10.1%)	288 (26.9%)	272 (25.4%)	
***Leisure-Time Physical Activity***						0.437
Sufficient	479 (44.8%)	44.1%	49.1%	47.2%	41.5%	
Insufficient	590 (55.2%)	55.9%	50.9%	52.8%	58.5%	
***Transport-Related Physical Activity***						<0.001 **
Sufficient	738 (69.0%)	80.0%	75.9%	60.8%	58.8%	
Insufficient	331 (31.0%)	20.0%	24.1%	39.2%	41.2%	
***Sleep***						0.390
Appropriate	664 (62.1%)	60.3%	69.4%	62.2%	61.8%	
Inappropriate	405 (37.9%)	39.7%	30.6%	37.8%	38.2%	
***Alcohol Use***						0.653
Yes	112 (10.5%)	10.5%	13.0%	11.1%	8.8%	
No	957 (89.5%)	89.5%	87.0%	88.9%	91.2%	
***Smoking Status***						0.013 *
Yes	141 (13.2%)	9.7%	9.3%	16.3%	16.5%	
No	928 (86.8%)	90.3%	90.7%	83.7%	83.5%	
***Dietary Behavior***						0.957
Yes	788 (73.7%)	74.1%	74.1%	74.3%	72.4%	
No	281 (26.3%)	25.9%	25.9%	25.7%	27.6%	

* *p* < 0.05; ** *p* < 0.001.

**Table 3:** Frequency of motorcycle use associated with overweight among Taiwanese adults.

Motorcycle Use	*n*	%	Odds of Being Overweight	
			Model 1	Model 2
			OR (95% CI)	
Q1	401	37.5%	1.00	1.00
Q2	108	10.1%	1.29 (0.84–1.99)	1.15 (0.72–1.85)
Q3	288	26.9%	1.17 (0.85–1.59) *	1.08 (0.77–1.52)
Q4	272	25.4%	1.60 (1.17–2.20) **	1.50 (1.06–2.13) *

CI: confidence interval; OR: odds ratio; Model 1 is an unadjusted model; Model 2 is adjusted for the gender, age, educational level, occupational type, marital status, living status, car ownership, and lifestyle behavior including transport-related physical activity, leisure-time physical activity, sleep, alcohol use, current smoking status, and dietary behavior; * *p* < 0.05; ** *p* < 0.001.
